# Hydrogen Peroxide Production by Streptococcus pneumoniae Results in Alpha-hemolysis by Oxidation of Oxy-hemoglobin to Met-hemoglobin

**DOI:** 10.1128/mSphere.01117-20

**Published:** 2020-12-09

**Authors:** Erin McDevitt, Faidad Khan, Anna Scasny, Courtney D. Thompson, Zehava Eichenbaum, Larry S. McDaniel, Jorge E. Vidal

**Affiliations:** a Department of Microbiology and Immunology, University of Mississippi Medical Center, Jackson, Mississippi, USA; b Department of Biology, Georgia State University, Atlanta, Georgia, USA; University of Arizona

**Keywords:** *Streptococcus pneumoniae*, alpha-hemolysis, hemoglobin, met-hemoglobin, oxidation

## Abstract

Streptococcus pneumoniae and other streptococci produce a greenish halo on blood agar plates referred to as alpha-hemolysis. This phenotype is utilized by clinical microbiology laboratories to report culture findings of alpha-hemolytic streptococci, including S. pneumoniae, and other bacteria. The alpha-hemolysis halo on blood agar plates has been related to the hemolytic activity of pneumococcal pneumolysin (Ply) or, to a lesser extent, to lysis of erythrocytes by S. pneumoniae-produced hydrogen peroxide. We investigated the molecular basis of the alpha-hemolysis halo produced by S. pneumoniae. Wild-type strains TIGR4, D39, R6, and EF3030 and isogenic derivative Δ*ply* mutants produced similar alpha-hemolytic halos on blood agar plates, while cultures of hydrogen peroxide knockout Δ*spxB* Δ*lctO* mutants lacked this characteristic halo. Moreover, in the presence of catalase, the alpha-hemolysis halo was absent in cultures of the wild-type (wt) and Δ*ply* mutant strains. Spectroscopic studies demonstrated that culture supernatants of TIGR4 released hemoglobin-bound heme (heme-hemoglobin) from erythrocytes and oxidized oxy-hemoglobin to met-hemoglobin within 30 min of incubation. As expected, given Ply hemolytic activity and that hydrogen peroxide contributes to the release of Ply, TIGR4Δ*ply* and Δ*spxB* Δ*lctO* isogenic mutants had significantly decreased release of heme-hemoglobin from erythrocytes. However, TIGR4Δ*ply* that produces hydrogen peroxide oxidized oxy-hemoglobin to met-hemoglobin, whereas TIGR4Δ*spxB* Δ*lctO* failed to produce oxidation of oxy-hemoglobin. Studies conducted with all other wt strains and isogenic mutants resulted in similar findings. We demonstrated that the so-called alpha-hemolysis halo is caused by the oxidation of oxy-hemoglobin (Fe^+2^) to a non-oxygen-binding met-hemoglobin (Fe^+3^) by S. pneumoniae-produced hydrogen peroxide.

**IMPORTANCE** There is a misconception that alpha-hemolysis observed on blood agar plate cultures of Streptococcus pneumoniae and other alpha-hemolytic streptococci is produced by a hemolysin or, alternatively, by lysis of erythrocytes caused by hydrogen peroxide. We noticed in the course of our investigations that wild-type S. pneumoniae strains and hemolysin (e.g., pneumolysin) knockout mutants produced the alpha-hemolytic halo on blood agar plates. In contrast, hydrogen peroxide-defective mutants prepared in four different strains lacked the characteristic alpha-hemolysis halo. We also demonstrated that wild-type strains and pneumolysin mutants oxidized oxy-hemoglobin to met-hemoglobin. Hydrogen peroxide knockout mutants, however, failed to oxidize oxy-hemoglobin. Therefore, the greenish halo formed on cultures of S. pneumoniae and other so-called alpha-hemolytic streptococci is caused by the oxidation of oxy-hemoglobin produced by hydrogen peroxide. Oxidation of oxy-hemoglobin to the nonbinding oxygen form, met-hemoglobin, might occur in the lungs during pneumococcal pneumonia.

## Abstract (precis)

There is a misconception that alpha-hemolysis observed on blood agar plate cultures of Streptococcus pneumoniae and other alpha-hemolytic streptococci is produced by a hemolysin or, alternatively, by lysis of erythrocytes caused by hydrogen peroxide. We noticed in the course of our investigations that wild-type S. pneumoniae strains and hemolysin (e.g., pneumolysin) knockout mutants produced the alpha-hemolytic halo on blood agar plates.

## OBSERVATION

Historically, Streptococcus pneumoniae and other streptococci of the viridians group are classified as alpha-hemolytic bacteria on the basis of a greenish halo that surrounds colonies when grown aerobically on blood agar plates (1, 2). This alpha-hemolytic activity has been related to the production of a hemolysin, which, in the case of S. pneumoniae strains, is referred as to pneumolysin (Ply) (3). An observation made that anaerobic cultures of alpha-hemolytic streptococci lack this greenish discoloration has linked the phenotype also to the lysis of erythrocytes by hydrogen peroxide that streptococci produce as a metabolic by-product (1). In this study, we investigated whether Ply or S. pneumoniae-produced hydrogen peroxide was responsible for alpha-hemolysis and identified the molecular basis of the phenotype.

Pneumolysin is encoded by *ply* (4), while hydrogen peroxide released in cultures of S. pneumoniae strains and alpha-hemolytic streptococci is a by-product of the metabolism of the enzymes pyruvate oxidase (SpxB) and lactate dehydrogenase (LctO) (5). We utilized different Δ*ply* mutants and S. pneumoniae TIGR4Δ*spxB* Δ*lctO* from previous publications (6–8) and prepared Δ*spxB* Δ*lctO* double mutants in three other backgrounds, vaccine serotype 19F strain EF3030 (9, 10), serotype 2 strain D39, and strain R6, and a new EF3030Δ*ply* mutant (11). We previously demonstrated by Western blotting and a hemoglobin release assay that the Ply knockout mutants do not produce Ply (6) and that TIGR4Δ*spxB* Δ*lctO* does not produce detectable levels of hydrogen peroxide in supernatants from Todd-Hewitt broth supplemented with yeast extract (THY broth) cultures incubated for 4 h (7).

**FIG 1:**
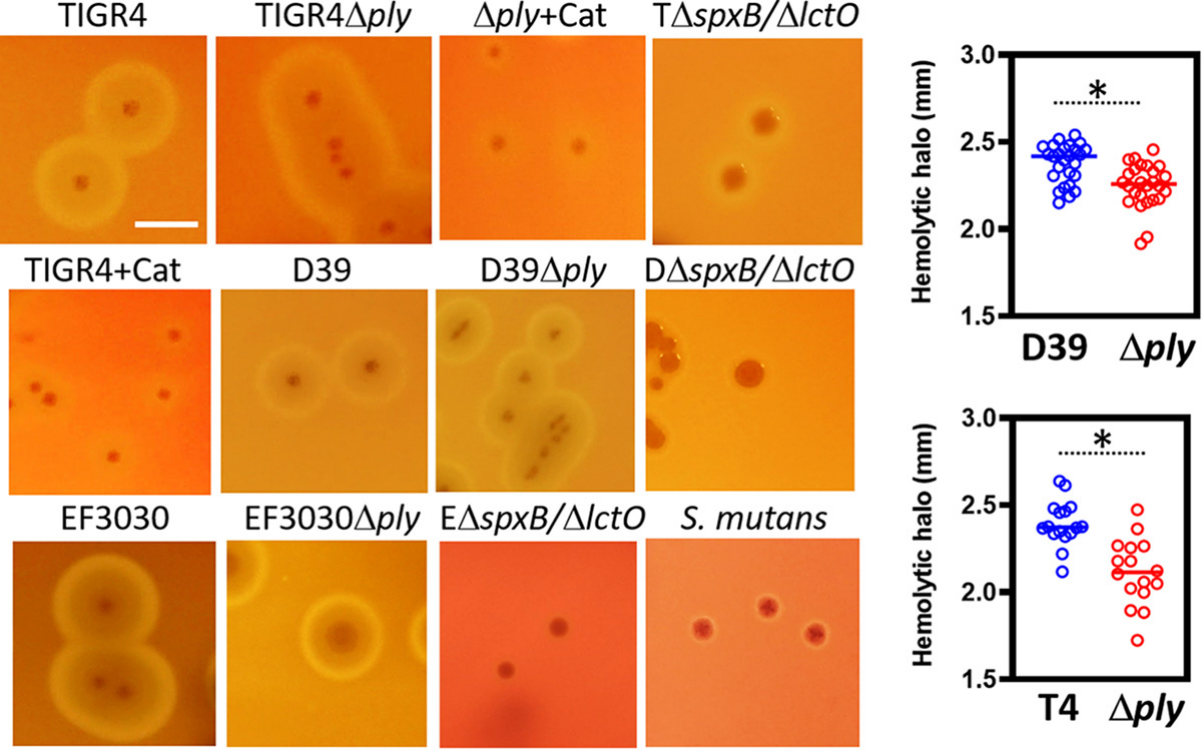
Hydrogen peroxide but not pneumolysin causes the alpha-hemolytic phenotype on blood agar plates. S. pneumoniae wt strains TIGR4 (T), D39 (D), and EF3030 (E) or Δ*ply* and Δ*spxB* Δ*lctO* mutant derivatives, or S. mutans strain ATCC 25175, were inoculated onto blood agar plates and incubated for 24 h at 37°C in a 5% CO_2_ atmosphere. Another set of plates were added with 400 U/ml of catalase (Cat) and then inoculated as described above. Plates were photographed with a Canon Rebel EOS T5 camera system, and digital pictures were analyzed. Phenotypes were confirmed at least three times. Bar, 2 mm. (Right) Hemolytic halos measured with ImageJ software for at least 25 colonies from images obtained from cultures on blood agar plates of D39 wt, D39Δ*ply*, TIGR4 (T4), or TIGR4Δ*ply*; unpaired Student's *t* test was performed to assess significance. *, *P* < 0.05.

S. pneumoniae strains were inoculated on blood agar plates containing 5% sheep blood, and plates were incubated at 37°C under aerobic conditions and a 5% CO_2_ atmosphere. Overnight cultures of strains TIGR4, D39, R6, and EF3030 showed the classic alpha-hemolytic halo surrounding colonies, whereas D39Δ*ply*, R6Δ*ply*, TIGR4Δ*ply*, and EF3030Δ*ply* mutant strains also showed an indistinguishable alpha-hemolysis halo (Fig. 1 and not shown). The median diameters of alpha-hemolysis halos produced by the D39 wild type (wt), TIGR4 wt, and EF3030 wt were very similar although statistically different from those produced by the respective *ply* mutant strains (Fig. 1, inset, and not shown). Blood agar plates with cultures of isogenic D39Δ*ply* produced alpha-hemolysis halos with a diameter of 2.26 mm (Fig. 1, inset). An additional TIGR4Δ*ply* mutant (AC4037) yielded a similar alpha-hemolysis halo (not shown) (12). In contrast, blood agar plates inoculated with TIGR4Δ*spxB* Δ*lctO* and three additional double Δ*spxB* Δ*lctO* mutants, R6Δ*spxB* Δ*lctO*, D39Δ*spxB* Δ*lctO*, and EF3030Δ*spxB* Δ*lctO*, completely lacked the alpha-hemolytic halo (Fig. 1). Blood agar plates of TIGR4Δ*spxB* Δ*lctO*, D39Δ*spxB* Δ*lctO*, and EF3030Δ*spxB* Δ*lctO* did not produce the alpha-hemolytic halo even after 72 h of incubation (not shown).

To confirm that hydrogen peroxide was responsible for the alpha-hemolytic halo, TIGR4 wt or its isogenic TIGR4Δ*ply* was inoculated on blood agar plates containing catalase (400 U/ml). As shown in Fig. 1, catalase inhibited alpha-hemolysis. Moreover, adding pure hydrogen peroxide to blood agar plates spanning the concentration produced by S. pneumoniae strains (i.e., 800, 80, and 8 μM) produced similar alpha-hemolytic halos on plates made of sheep or horse blood (not shown). Other streptococci, including S. mutans, are not hemolytic when grown on blood agar plates (1, 2). Colonies of S. mutans strain ATCC 25175 on blood agar plates resembled those of the S. pneumoniae isogenic hydrogen peroxide knockout mutants (Fig. 1). Similarly to S. pneumoniae and other streptococci, S. mutans harbors a putative α-hemolysin (13). S. mutans lacks production of detectable hydrogen peroxide in the supernatant when grown in aerobic cultures, and it is highly susceptible to hydrogen peroxide produced by alpha-hemolytic oral streptococci (14, 15). Altogether, this evidence indicates that hydrogen peroxide but not the hemolysin Ply caused the alpha-hemolytic phenotype observed in aerobic cultures of S. pneumoniae strains.

Erythrocytes carry hemoglobin that reversibly binds oxygen through a penta-coordinate heme molecule containing ferrous iron (Fe^+2^), known as oxy-hemoglobin (16). When hemoglobin is released from erythrocytes, heme-hemoglobin can be observed by optical spectroscopy at ∼415 nm (16–18). This region is known as the Soret region peak and represents heme-hemoglobin, while oxy-hemoglobin is characterized by two absorption peaks of ∼540 and ∼570 nm (17, 18). Oxy-hemoglobin (Fe^+2^) is autoxidized to met-hemoglobin (Fe^+3^) or oxidized by radicals such as hydrogen peroxide (17, 18), inducing spectral changes, i.e., flattening the oxy-hemoglobin absorbance peaks. S. pneumoniae produces and releases an abundance of hydrogen peroxide into the culture supernatant that intoxicates human cells (19) or that rapidly kills Staphylococcus aureus strains and other bacterial species (7, 20). Hydrogen peroxide is a by-product of the metabolism of two different enzymes, pyruvate oxidase (SpxB) and lactate dehydrogenase (LctO) (5).

**FIG 2:**
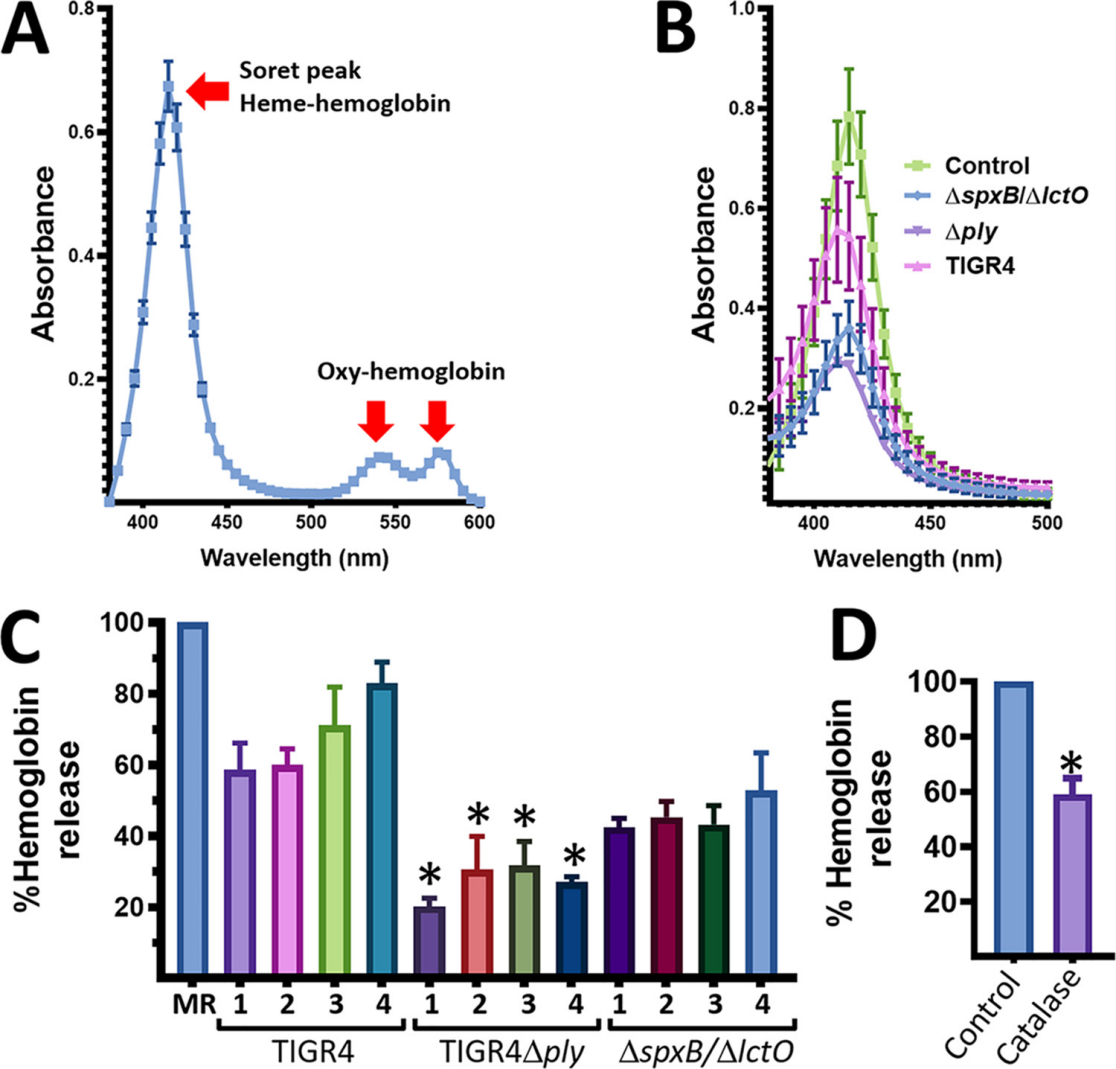
Heme-hemoglobin release by S. pneumoniae strains. (A) Suspension (3%) of sheep erythrocytes was lysed, centrifuged at 300 × *g* for 5 min, and incubated for 30 min at 37°C in a 5% CO_2_ atmosphere; the spectrum was obtained using a spectrophotometer Omega BMG LabTech (Thermo Fisher). (B and C) TIGR4 wt, TIGR4Δ*ply*, or TIGR4Δ*spxB* Δ*lctO* was inoculated in THY broth (pH 7.0) and incubated for 4 h at 37°C in a 5% CO_2_ atmosphere. (D) TIGR4Δ*ply* was incubated in THY broth alone or with catalase (200 U/ml) for 4 h. Bacterium-free supernatants were harvested by centrifugation at 13,000 × *g* for 5 min, and equal volumes were incubated with a 3% suspension of sheep erythrocytes for 30 min at 37°C. After pelleting down the erythrocytes as described above, the hemoglobin-containing supernatant was collected. (B) The UV-visible absorption spectrum was obtained at the 4-h time point. Maximum heme-hemoglobin release (C) or that released by an untreated control (D) was set to 100%, and the percent release by culture supernatants was calculated. Error bars represent the standard errors of the means calculated using data from at least three independent experiments. Student *t* test (*, *P* < 0.05) analysis was performed to compare Soret absorbances at 415 nm generated by the wt and isogenic mutant at the same time point.

To further investigate the molecular basis of the alpha-hemolytic phenotype, we utilized a modified hemoglobin release assay that, when coupled with optical spectroscopy, allowed us to quantify the release of heme-hemoglobin and to observe the oxidation of oxy-hemoglobin to met-hemoglobin. As a control of heme-hemoglobin release and the presence of oxy-hemoglobin, we obtained the UV-visible absorption spectra of a 3% suspension of sheep erythrocytes that had been lysed with an equal volume of water or lysed with 0.1% final concentration of saponin (Fig. 2A and not shown). After centrifugation of the lysed erythrocyte suspension at 300 × *g* for 5 min, no red blood cells were visible in the bottom; therefore, this was considered the maximum heme-hemoglobin released. As expected, three characteristic peaks were observed. The Soret peak, for which the wavelength of maximum absorption was 415 nm and its absorbance was set as 100% hemoglobin release (Fig. 2A), and two oxy-hemoglobin peaks at 540 and 570 nm (Fig. 2A). Similar peaks were observed when hemoglobin was released from sheep or horse erythrocytes with saponin (not shown). To investigate the release of heme-hemoglobin, S. pneumoniae strains were inoculated at similar densities of ∼5 × 10^7^ CFU/ml in THY broth (pH 7) and incubated at 37°C in a 5% CO_2_ atmosphere for 1, 2, 3, or 4 h. Bacterium-free supernatants were harvested and then incubated with equal volumes of a 3% suspension of sheep erythrocyte at 37°C in a 5% CO_2_ atmosphere for 30 min, after which, the treated erythrocyte suspensions were centrifuged at 300 × *g* for 5 min to collect supernatants. Experiments presented below were conducted with TIGR4 wt and its isogenic mutants. We also performed similar experiments using D39, R6, and EF3030 wt strains and their isogenic mutants, with essentially similar results (not shown).

The Soret peak of heme-hemoglobin released in the control (Fig. 2B) represented 100% of heme-hemoglobin released (Fig. 2C). A time course study demonstrated that TIGR4 released ∼60% of heme-hemoglobin as soon as 1 h postinoculation (Fig. 2B and C) and produced, after 4 h of incubation, a Soret peak representing ∼85% of heme-hemoglobin released compared to the maximum heme-hemoglobin released in the control (Fig. 2C). As expected given that hydrogen peroxide contributes to release of Ply into the supernatant (21), hemoglobin released by TIGR4Δ*spxB* Δ*lctO* after 4 h of incubation was ∼50% of that released by the wt strain (Fig. 2B and C). In contrast to the TIGR4 strain and TIGR4Δ*spxB* Δ*lctO*, the isogenic Ply knockout mutant TIGR4Δ*ply* induced the release of <30% heme-hemoglobin compared to that by the control. This residual release of hemoglobin was in part caused by hydrogen peroxide activity in the supernatants, since it was significantly reduced in cultures of TIGR4Δ*ply* incubated with catalase (200 U/ml) (Fig. 2D). Because a mutation in *ply* renders the strain unable to lyse erythrocytes, all but Δ*ply* mutant strains produce the so-called alpha-hemolytic phenotype; these results further support that Ply-associated hemolytic activity is not responsible for the alpha-hemolysis phenotype observed in blood agar plates.

**FIG 3:**
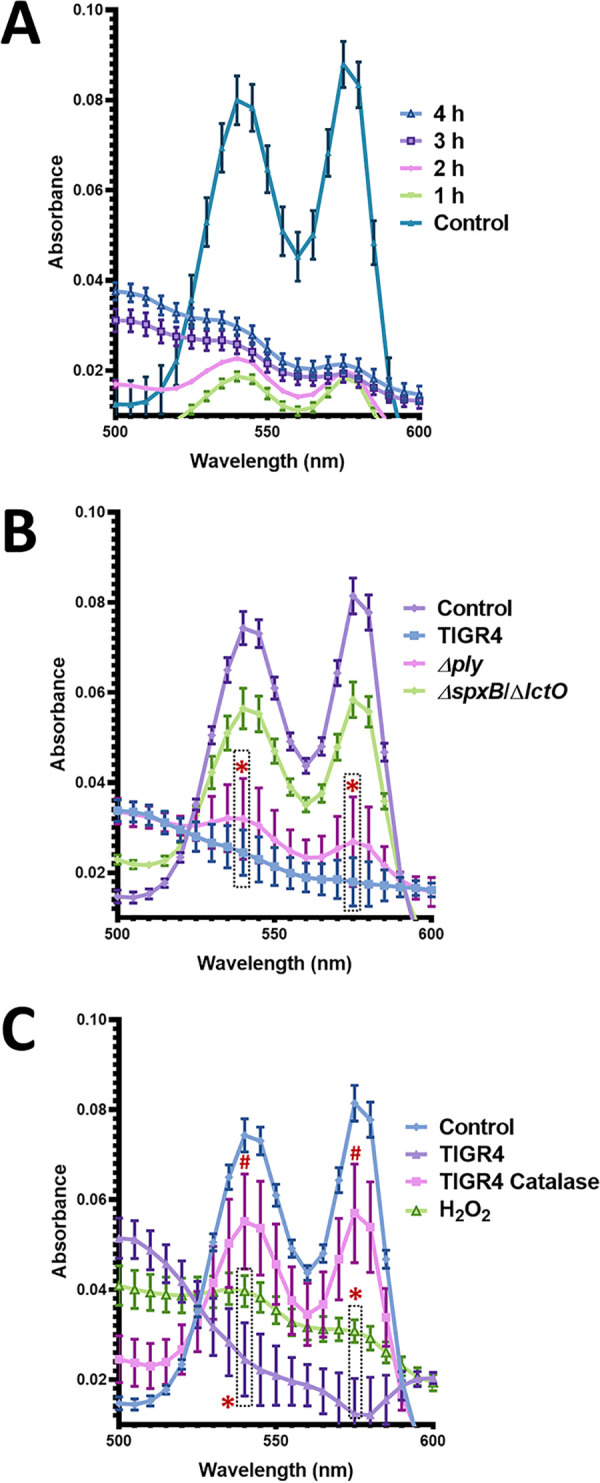
Oxy-hemoglobin is oxidized to met-hemoglobin by S. pneumoniae-produced hydrogen peroxide. (A) Suspension (3%) of sheep erythrocytes was mixed with equal volumes of cell-free culture supernatants of strain TIGR4 wt that had been grown as described for Fig. 2B for the indicated times. The mixture was incubated for 30 min at 37°C in a 5% CO_2_ atmosphere. As a control, erythrocytes were lysed and incubated under the same conditions. The absorbance spectra were then obtained using a spectrophotometer Omega BMG LabTech (Thermo Fisher). (B) TIGR4 wt, TIGR4Δ*ply*, or TIGR4Δ*spxB* Δ*lctO* was inoculated in THY broth (pH 7.0) and incubated for 4 h at 37°C in a 5% CO_2_ atmosphere. Bacterium-free supernatants were harvested by centrifugation at 13,000 × *g* for 5 min, and equal volumes were incubated for 30 min at 37°C with hemoglobin-containing erythrocytes lysates. Hemoglobin-containing supernatants were collected, and the UV-visible absorption spectra were obtained. (C) Oxy-hemoglobin-containing lysates (control) were incubated for 30 min at 37°C with H_2_O_2_ (880 μM), TIGR4 wt supernatant harvested as described above, or TIGR4 wt supernatant and catalase (200 U/ml). Error barsrepresent the standard errors of the means calculated using data from at least three independent experiments. Student *t* test (*, *P* < 0.05) analysis was performed to compare the oxy-hemoglobin absorbance peaks (540 nm and 570 nm) generated by the untreated hemoglobin-containing lysate control against that generated by incubation with supernatants from TIGR4 wt, TIGR4Δ*spxB* Δ*lctO*, TIGR4Δ*ply* strain (B), or H_2_O_2_ (C). #, *P* < 0.05 comparing TIGR4 wt incubated with catalase against TIGR4 wt and H_2_O_2._

Oxy-hemoglobin can react with reactive oxygen species, including hydrogen peroxide, to produce met-hemoglobin, the non-oxygen-binding form of hemoglobin (22, 23). To assess the presence of met-hemoglobin, we evaluated the oxy-hemoglobin peaks in hemoglobin preparations incubated with S. pneumoniae supernatants. Oxy-hemoglobin peaks were clearly observed in the control preparation (Fig. 3A) but were completely flattened when culture supernatants of the TIGR4 strain obtained after 3 or 4 h of incubation were incubated with the suspension of erythrocytes for an additional 30-min period. This change in the absorption pattern of oxy-hemoglobin was compatible with the oxidation of oxy-hemoglobin to met-hemoglobin (22). Note that autoxidation of oxy-hemoglobin to met-hemoglobin did not occur within the 30-min incubation of the assay, since the oxy-hemoglobin peaks were observed. Because culture supernatants from TIGR4Δ*ply* or TIGR4Δ*spxB* Δ*lctO* did not contain heme-hemoglobin at the same level as those from TIGR4, we could not evaluate the oxidation of oxy-hemoglobin in these isogenic mutant strains using the modified hemoglobin release assay.

To further confirm whether oxy-hemoglobin is oxidized to met-hemoglobin by hydrogen peroxide produced in culture supernatants of TIGR4Δ*ply* but not in supernatants of hydrogen peroxide knockout mutant TIGR4Δ*spxB* Δ*lctO*, we incubated preparations of oxy-hemoglobin that had been previously released from erythrocytes, as mentioned earlier, with culture supernatants of TIGR4 or isogenic mutants. We reasoned that if hydrogen peroxide present in culture supernatants was responsible for the oxidation of oxy-hemoglobin, then having oxy-hemoglobin already as a substrate would allow us to observe such a reaction. As expected, supernatants from 4-h cultures of the wt strain that were incubated for 30 min with the oxy-hemoglobin preparation converted oxy-hemoglobin to met-hemoglobin (Fig. 3B). Supernatants from the isogenic TIGR4Δ*ply* significantly oxidized oxy-hemoglobin to met-hemoglobin, indicating that oxidation occurred due to the hydrogen peroxide activity retained by the *ply* knockout mutant. Confirming this hypothesis, oxy-hemoglobin was observed almost intact after a 30-min incubation with supernatants of the isogenic TIGR4Δ*spxB* Δ*lctO*, indicating that met-hemoglobin was produced by hydrogen peroxide secreted into the culture supernatant. Oxidation of oxy-hemoglobin did not occur in supernatants of the wt strain treated with catalase (200 U/ml), but oxy-hemoglobin was oxidized to met-hemoglobin when the oxy-hemoglobin preparations were treated with H_2_O_2_ (880 μM) at a similar concentration to that produced in culture supernatants of S. pneumoniae strains (7, 21, 24) (Fig. 3C). Oxidation of hemoglobin to met-hemoglobin was observed using horse erythrocytes and when releasing hemoglobin from erythrocytes using water or saponin (not shown).

In conclusion, we demonstrated in this study that the so-called alpha-hemolysis phenotype observed on blood agar plates when incubated under aerobic conditions is an oxidative reaction caused by S. pneumoniae-produced hydrogen peroxide that converts oxy-hemoglobin to met-hemoglobin.
